# Unified Multi-Modal Object Tracking Through Spatial–Temporal Propagation and Modality Synergy

**DOI:** 10.3390/jimaging11120421

**Published:** 2025-11-22

**Authors:** Jiajia Wu, Haorui Zuo, Yuxing Wei, Meihui Li, Jianlin Zhang

**Affiliations:** 1 State Key Laboratory of Optical Field Manipulation Science and Technology, Institute of Optics and Electronics, Chinese Academy of Sciences, Chengdu 610209, China; wujiajia17@mails.ucas.ac.cn (J.W.); zuohaorui@ioe.ac.cn (H.Z.); weiyuxing@ioe.ac.cn (Y.W.); limeihui@ioe.ac.cn (M.L.); 2 National Laboratory on Adaptive Optics, Chengdu 610209, China; 3 Institute of Optics and Electronics, Chinese Academy of Sciences, Chengdu 610209, China

**Keywords:** unified multi-modal tracking, spatial-temporal propagation, modality synergy, adaptive perception fusion

## Abstract

Multi-modal object tracking (MMOT) has received widespread attention for the ability to overcome single-sensor perception limitations. However, existing methods encounter several critical challenges. Representation learning and generalization capabilities of models are constrained by the inherent heterogeneity of cross-task multi-modal data and inter-modal synergy imbalance. Particularly, in dynamically changing complex scenarios, the reliability and stability of data significantly degrade, further exacerbating the difficulty in multi-modal consistent perception and aggregation. To tackle the above issues, we propose SMUTrack, a unified framework with global shared parameters integrating three downstream MMOT tasks. SMUTrack implements a batch merging-and-splitting alternating strategy, coupled with multi-task joint training, to establish latent correlations across inter- and intra-task modalities, effectively avoiding over-reliance on certain modalities. Concurrently, we design a hierarchical modality synergy and reinforcement (HMSR) module, and a gated fusion and context awareness (GFCA) module to enable progressive multi-modal information exchange and integration, yielding the more discriminative and robust multi-modal representation. More importantly, we introduce a spatial–temporal information propagation (SIP) mechanism, which synchronously learns object trajectory cues and appearance variations to effectively build contextual relationships in long-term video tracking. Experimental results definitively validate the outstanding performance of SMUTrack on mainstream MMOT datasets, exhibiting its powerful adaptability to various MMOT tasks.

## 1. Introduction

Single object tracking (SOT) [1,2,3,4,5], a fundamental task in computer vision, aims to continuously and accurately locate a specific object in a video sequence while capturing its bounding box and motion trajectory. Its breakthroughs are crucial for various applications such as autonomous driving [6,7], intelligent monitoring [8,9], and human–computer interaction [10,11]. While RGB-based object tracking technology has made significant progress, it still struggles with various interferences in complex open-world environments, including low illumination [12], occlusion [13], similar appearance [14], motion blur [15], etc. In these extreme conditions, only relying on RGB features is insufficient to comprehensively capture the appearance and motion state of objects, severely limiting tracking performance.

**Figure 1:**
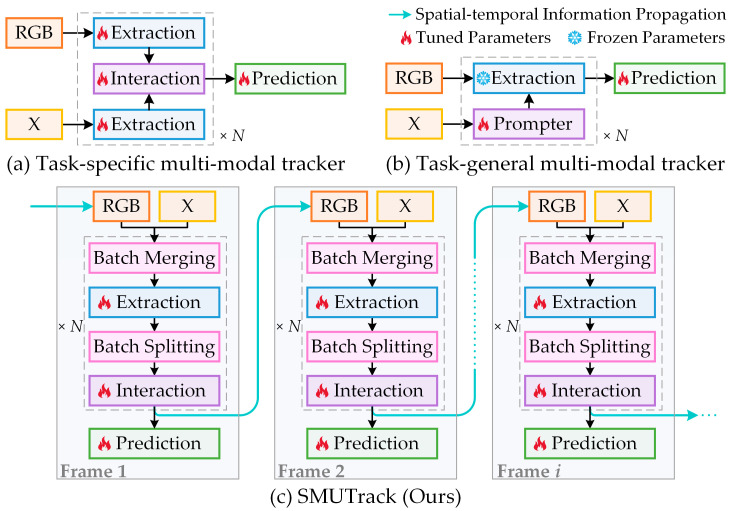
Comparison of multi-modal object tracking frameworks. (**a**) Task-specific multi-modal trackers primarily adopt symmetric dual-encoding architectures, tailored for a single RGB-X task. (**b**) Task-general multi-modal trackers typically treat X modality as auxiliary and introduce prompt learning to accommodate diverse multi-modal tracking tasks within a unified framework. (**c**) Our proposed SMUTrack is a powerful unified model that introduces batch operations and spatial–temporal information propagation in addition to enhancing inter-modal collaboration, hence diminishing cross-task and cross-modality barriers by one training session.

Multi-modal object tracking (MMOT) [16,17,18,19,20] offers an effective solution to break through the performance bottleneck of single-modal object tracking (SMOT). The key distinction lies in that MMOT introduces a supplementary modality (X) such as depth (D) [21], thermal (T) [22], and event (E) [23], and explores the complementary relationship between RGB and X modalities to strengthen the feature representation by deploying additional multi-modal feature interaction and fusion process, thereby mitigating reliance on single-modal data (typically RGB). Nevertheless, previous MMOT trackers generally present several critical challenges. Most works [13,15,24] are devoted to designing a network architecture for a specific RGB-X tracking task (Figure 1a) without considering potential modality relationships across tasks. Although some studies [25,26] have proposed general models (Figure 1b), parameters need to be retrained for different RGB-X tasks. These general models typically prioritize RGB and regard X modality as auxiliary, which leads to the unique semantics of X modality to be underutilized. Overall, inter-task independence and intra-task synergy imbalance make it difficult for models to overcome the inherent heterogeneity of various modality data and learn effective global representations, thereby limiting the generalization ability. Moreover, tracking is a dynamic process that frequently encounters data quality degradation as real-world scenarios continuously change. This necessitates effectively identifying and suppressing hazardous information, placing higher demands on cross-modal synergy perception. Simultaneously, this process may involve object occlusion and deformation, resulting in incomplete or invalid perception. However, existing methods focus more on synchronous fusion of multi-modal features, while neglecting explicit modeling for long-term context and spatial–temporal dependencies in video sequences, which makes trackers struggle to handle such scenarios.

To solve the above issues, this paper proposes a unified multi-modal object tracking framework, named SMUTrack, which comprehensively excavates spatial–temporal information and inter-modal synergy relationships in video sequences to promote tracking robustness and generalization ability. Different from treating X modality as prompt cues, SMUTrack regards RGB and X modalities equally by alternately performing batch merging and batch splitting, allowing RGB and diverse X modalities to entirely share an encoder, as shown in Figure 1c. SMUTrack can achieve seamless adaptation to three downstream MMOT tasks (i.e., RGB-D, RGB-T, and RGB-E) with only one training session. Specifically, in terms of spatial–temporal modeling, we design a spatial–temporal information propagation (SIP) mechanism that integrates temporal prompt learning into multi-modal representation learning. This mechanism leverages historical temporal prompts to guide the temporal state generation and object feature enhancement in the current frame, thus facilitating the flow of temporal information across frames. To dynamically adapt to object appearance variations during tracking, the SIP mechanism further introduces a template update strategy with long-term and short-term memory. Regarding multi-modal feature exchange and integration, we first construct a hierarchical modality synergy and reinforcement (HMSR) module, which embeds mamba synergy prompt blocks (MSPBs) into three stages of the transformer encoder. This design enables RGB and X modality features to interact fully at different levels, progressively enhancing the feature representation capabilities of each modality. To achieve deep integration of multi-modal features, we then develop a gated fusion and context awareness (GFCA) module. In this module, the gated fusion unit (GFU) comprehensively evaluates the importance of each modality based on image tokens, adaptively selecting and fusing effective information. Meanwhile, the context awareness unit (CAU) utilizes mamba to adequately integrate multi-modal fusion features from different levels, obtaining representations with global contextual information.

The primary contributions in this article can be encapsulated as follows.

(1) A unified multi-modal tracking framework, SMUTrack, is proposed to fully explore spatial–temporal information and inter-modal synergy relationships. SMUTrack supports three MMOT tasks (e.g., RGB-D, RGB-T, and RGB-E) in parallel, and the four modalities share a same encoding backbone entirely.

(2) We propose a SIP mechanism, which incorporates temporal evolution and templates updating to explicitly model long-term contextual relationships in video sequences. This improves tracking robustness in challenging scenes, typically involving occlusions and changing appearance.

(3) We establish an HMSR module and a GFCA module to accomplish progressive interaction and adaptive perception fusion for cross-modal features, remarkably boosting the model’s ability in representational learning.

(4) Comprehensive experimental results revealed that SMUTrack attains cutting-edge performance on five mainstream MMOT datasets, exhibiting powerful adaptability to diverse tasks and environments.

The subsequent content is arranged as follows. Section 2 reviews and analyzes relevant research on multi-modal object tracking and spatial–temporal modeling. Section 3 elaborates the proposed SMUTrack. Section 4 delivers experimental results and discussion. Section 5 summarizes the entire work and prospects future research directions.

## 2. Related Works

### 2.1. Multi-Modal Object Tracking

As an extension of object tracking, multi-modal object tracking (MMOT) provides richer semantic clues for object representation in complex scenes by integrating complementary information from RGB and X modalities (e.g., depth, thermal, and event). It effectively copes with challenges such as occlusion, extreme weather, and drastic appearance changes. Existing MMOT methods can be categorized broadly into two types (Table 1): (1) Task-specific methods, which design independent network structures and functional modules tailored to specific RGB-X data. For instance, AMATrack [27] proposes an asymmetric mixed attention module for RGB-D task to build intra-modal and inter-modal relations. AMNet [17] achieves spatial alignment and fusion of RGB-T features through a mutual-interacted spatial alignment (MSA) module and an information matching fusion (IMF) module. GMMT [28] employs a generative model to augment RGB-T information fusion. RT-MDNet [23] introduces a cross-modality transformer for RGB-E tasks, enabling efficient fusion of visible and event data. (2) Task-general methods, which aim to make a single model compatible with multiple RGB-X tasks. For example, ViPT [25] incorporates prompt learning to streamline auxiliary modalities into a few visual prompts for adapting various MMOT tasks. SDSTrack [26] employs a lightweight adapter to transfer the feature extraction capability of the pre-trained model to other modalities. However, these methods still require separate training parameters for different MMOT tasks, resulting in high training costs and poor generalization capabilities. To address this issue, Un-Track [29] proposes a tracker that learns a common latent space using low-rank factorization and reconstruction techniques, requiring only a single parameter set for any MMOT task. Nevertheless, treating X modalities as prompts tends to make the fusion feature dominated by RGB modality, which hinders information interaction and complementary fusion between modalities. To overcome the above issues regarding modality synergy imbalance and limited generalization ability, this paper proposes a straightforward yet effective unified MMOT framework, called SMUTrack. SMUTrack allows any modality (i.e., RGB, depth, thermal, and event) to share the same encoder. It embeds multiple mamba synergy prompt blocks (MSPBs) into the transformer encoder, achieving the relationship modeling and information flow of multi-modal features during the encoding process. Additionally, SMUTrack integrates gated fusion units (GFUs) and context awareness units (CAUs) to progressively extract fusion features with strong representational capability.

### 2.2. Spatial–Temporal Modeling

Spatial–temporal information is crucial for object tracking. It can accurately capture the trajectory and appearance change trends of moving objects, and effectively deal with challenges such as object occlusion, temporary disappearance, and deformation. Therefore, related studies have extensively explored the application of spatial–temporal information in object tracking. These methods primarily focus on two aspects (Table 2): (1) They update appearance representations [30,31,32,33], which typically adopt template update strategies to capture real-time changes in object appearance. (2) They emphasize temporal correlation modeling [34,35,36]. For instance, TCTrack [36] proposes an online temporally adaptive convolution and an adaptive temporal transformer to enhance spatial features and refine similarity maps by leveraging temporal information. AQATrack [34] introduces queries to learn spatial–temporal information, and proposes a spatial–temporal information fusion module for aggregating static appearance and instantaneous changes. ARTrack [3] is a time-autoregressive framework for modeling the sequential evolution of trajectories, using previous tracking results as subsequent spatial–temporal cues. These studies confirm the significant role of spatial–temporal cues. However, most existing MMOT methods focus on exploring inter-modal complementarity and fail to fully utilize spatial–temporal information. Moreover, traditional sparse template update approaches struggle to establish stable spatial–temporal associations between search frames and template frames. Therefore, we propose a spatial–temporal information propagation (SIP) mechanism, and it introduces a set of learnable and autoregressive modal-specific temporal tokens, combined with a temporal guided attention mechanism (TGAM) and a template update strategy with long-term and short-term memory. This mechanism holistically associates spatial–temporal information into each stage of the MMOT framework.

## 3. Methods

**Figure 2:**
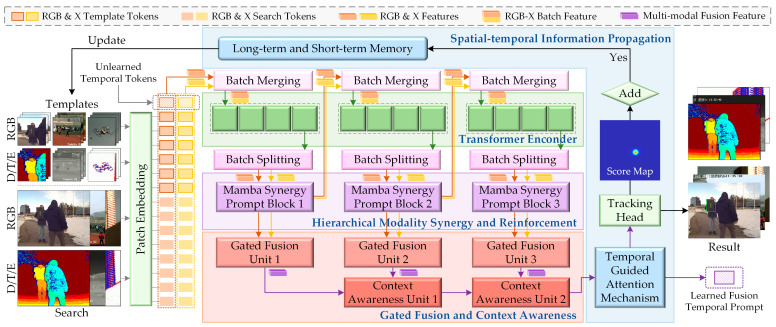
Overall architecture of the proposed SMUTrack. Diverse modality images are first converted into token embeddings and fed into a transformer encoder alongside temporal tokens to extract multi-modal features and temporal prompts. Then, an HMSR module and a GFCA module progressively interact and integrate multi-modal spatial–temporal information. Finally, a SIP mechanism optimizes the fusion feature for prediction, and updates templates.

In this paper, we elaborate a unified multi-modal tracker by leveraging spatial–temporal propagation and modality synergy, termed SMUTrack. The tracker can simultaneously adapt to three downstream MMOT tasks (i.e., RGB-D, RGB-T, and RGB-E) with one training parameter set. As illustrated in Figure 2, the core architecture of SMUTrack comprises a transformer encoder, a hierarchical modality synergy and reinforcement (HMSR) module, a gated fusion and context awareness (GFCA) module, and a spatial–temporal information propagation (SIP) mechanism. These components are designed to deeply exploit inter-task and intra-task modality correlations, as well as spatial–temporal prompts in video sequences, thereby achieving robust tracking performance.

### 3.1. Feature Encoding

Different from most existing tracking methods that use X-modality (X∈{D,T,E}) as auxiliary input, our approach alternately performs batch merging and batch splitting for multi-modal feature extraction and interaction. This design endows RGB and X modalities with equal importance, effectively preventing dominance by any single modality and establishing intra-task modality relationships. Simultaneously, batch merging processing can significantly improve feature encoding efficiency. Specifically, given a pair of RGB-X search frames $\{S_{RGB}^{i},S_{X}^{i}\}\in {\mathbb{R}}^{3\times H_{S}\times W_{S}}$ and M pairs of template frames ${\left\{T_{RGB}^{m},T_{X}^{m}\right\}}_{m=1}^{M}\in {\mathbb{R}}^{3\times H_{T}\times W_{T}}$ for frame i, all frames are first projected into P×P patch sequences and flattened through embedding layers, obtaining search frame tokens $\{{\hat{S}}_{RGB}^{i},{\hat{S}}_{X}^{i}\}\in {\mathbb{R}}^{N_{s}\times D}$ and template frame tokens $\{{\hat{T}}_{RGB}^{m},{\hat{T}}_{X}^{m}\}\in {\mathbb{R}}^{N_{T}\times D}$ with position embeddings. $D=3P^{2}$ is embedding dimension, and $N_{S}=H_{S}W_{S}/P^{2}$, $N_{T}=H_{T}W_{T}/P^{2}$ are token numbers. Then, we concatenate the token embeddings from the template and search frames with the introduced modal-specific temporal tokens $\tau_{RGB}^{i}$, $\tau_{X}^{i}\in {\mathbb{R}}^{1\times D}$ (details in Section 3.2), so that

(1) $$H_{RGB}^{i,0}=[\tau_{RGB}^{i},{\hat{T}}_{RGB}^{1},\ldots ,{\hat{T}}_{RGB}^{M},{\hat{S}}_{RGB}^{i}]\in {\mathbb{R}}^{\left(1+MN_{Z}+N_{S}\right)\times D},$$

(2) $$H_{X}^{i,0}=[\tau_{X}^{i},{\hat{T}}_{X}^{1},\ldots ,{\hat{T}}_{X}^{M},{\hat{S}}_{X}^{i}]\in {\mathbb{R}}^{\left(1+MN_{Z}+N_{S}\right)\times D}$$

Then, $H_{RGB}^{i,0}$ and $H_{X}^{i,0}$ are merged along the batch dimension and form the final token embedding $H_{bm}^{i,0}=[H_{RGB}^{i,0};H_{X}^{i,0}]\in {\mathbb{R}}^{2\times N\times D}$, $N=1+MN_{Z}+N_{S}$, which is fed into the transformer encoder to synchronously extract the features of RGB and X modalities, and the corresponding modality-specific temporal prompts.

Furthermore, we co-train multiple MMOT tasks in a unified process by standardizing the quantity of different multi-modal data per batch. This enables the feature encoder to more effectively learn inter-task modality correlations. Through joint optimization of inter-task and intra-task modality interactions, the task adaptability and robustness of the model are comprehensively improved.

### 3.2. Spatial–Temporal Information Propagation

**Figure 3:**
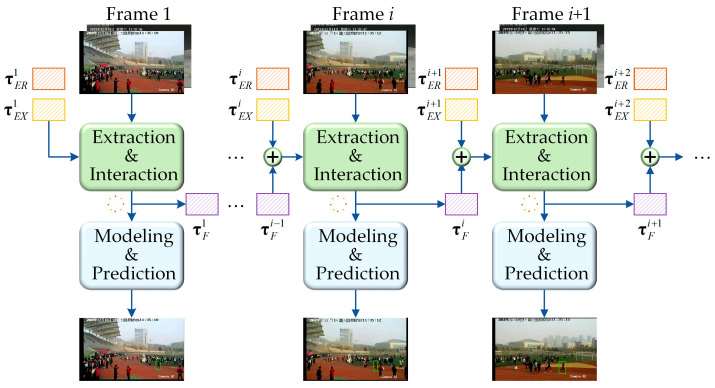
Spatial–temporal information propagation in SMUTrack. Our tracking pipeline introduces modality-specific temporal tokens and progressively learns a temporal prompt during feature extraction and interaction, propagating it to the next frame.

Existing multi-modal object trackers predominantly emphasize cross-modal feature interaction while neglecting the inherent spatial–temporal properties in video tracking. When confronted with challenging scenarios involving incomplete or failed perception, such as occlusion or dramatic appearance variations, tracking performance significantly deteriorates. Therefore, we propose a spatial–temporal information propagation (SIP) mechanism that effectively captures inter-frame dependencies in long-sequence tracking. Our tracking pipeline can handle arbitrary-length RGB-X videos, as illustrated in Figure 3. Specifically, learnable empty temporal tokens $\tau_{ER}^{1}$ and $\tau_{EX}^{1}$ are first embedded into the multi-modal tokens of the initial frame (refer to Equations (1) and (2)). Then, $H_{bm}^{1,0}$ is obtained and processed at the visual encoder and interaction layer to establish intrinsic spatial–temporal relationships, thereby learning modality-specific temporal prompts, ultimately producing a learned fusion temporal prompt $\tau_{F}^{1}\in {\mathbb{R}}^{1\times D}$. These prompts carry clues about the object trajectory. The learned prompt $\tau_{F}^{i}$ from the current frame serves as historical context for the next frame, enabling temporal information to flow seamlessly and gradually across frames, and the process can be formally expressed as,

(3) $$\tau_{RGB}^{i+1}=\tau_{ER}^{i+1}+\tau_{F}^{i},\;\tau_{ER}^{i+1}\in {\mathbb{R}}^{1\times D},$$

(4) $$\tau_{X}^{i+1}=\tau_{EX}^{i+1}+\tau_{F}^{i},\;\tau_{EX}^{i+1}\in {\mathbb{R}}^{1\times D}$$

where $\tau_{ER}^{i+1}$ and $\tau_{EX}^{i+1}$ are the initialized learnable temporal tokens for RGB and X modalities at frame i+1, respectively. We designed a temporal guided attention mechanism (TGAM) to further improve the temporal and trajectory perception of the final fusion feature. Specifically, TGAM calculates an attention weighting $W_{\tau }^{i}$ for the search region feature using the temporal prompt $\tau_{F}^{i}$ of the current frame,

(5) $$W_{\tau }^{i}={\tilde{S}}_{F}^{i}\cdot {\left(\tau_{F}^{i}\right)}^{⊤},\;W_{\tau }^{i}\in {\mathbb{R}}^{N_{S}\times 1}$$

where (⋅) is matrix multiplication. ${\tilde{S}}_{F}^{i}\in {\mathbb{R}}^{N_{S}\times D}$ represents the fusion feature of the search region. See Section 3.4 for calculation ${\tilde{H}}_{F}^{i}=[\tau_{F}^{i},{\tilde{T}}_{F}^{i},{\tilde{S}}_{F}^{i}]$. The weighting $W_{\tau }^{i}$ comprehensively analyzes the association between each token in the search region and the temporal prompt in a pixel-by-pixel manner. At the end, we introduce a residual connection to generate feature ${\hat{S}}_{F}^{i}$ for the tracking prediction head,

(6) $${\hat{S}}_{F}^{i}={\tilde{S}}_{F}^{i}+{\tilde{S}}_{F}^{i}⊗W_{\tau }^{i}$$

where ⊗ is element-wise multiplication. This operation can effectively preserve the discriminative details about the original feature. Overall, the introduction of modality-specific temporal tokens enables spatial–temporal interactions throughout the entire tracking pipeline, establishing a global topological relationship among historical trajectories, template regions, and search regions. This remarkably augments the tracker’s capacity for continuous object tracking.

To cope with drastic changes in object appearance, we design a dynamic template update strategy featuring long-term and short-term memory. Specifically, we build a historical template library and use the confidence score map from the tracking prediction head to ascertain whether to add the present tracking frame into the library, with a threshold of 0.65. To ensure the reliability of tracking templates while accommodating long-term evolution in object status, we select an original, a middle, and a latest template frame from this library as new templates for subsequent frame tracking.

### 3.3. Hierarchical Modality Synergy and Reinforcement

Effective cross-modal feature interaction is pivotal for improving tracking performance. It enables trackers to better understand scene information across different modalities, extracting the essence while discarding the irrelevant. To this end, we propose a hierarchical modality synergy and reinforcement (HMSR) module. This module deeply excavates the intrinsic correlation between RGB and X modality features through the mamba synergy prompt block (MSPB), achieving comprehensive interaction and complementary reconstruction of cross-modal information. Specifically, we divide the transformer encoder into three feature extraction stages, low, medium, and high levels, and embed MSPB at the end of each stage. By leveraging the efficient sequence modeling capability of mamba and the cross-modal synergy prompts, MSPB achieves deep interaction and enhancement of multi-modal features, greatly boosting the representation learning ability of the encoder.

**Figure 4:**
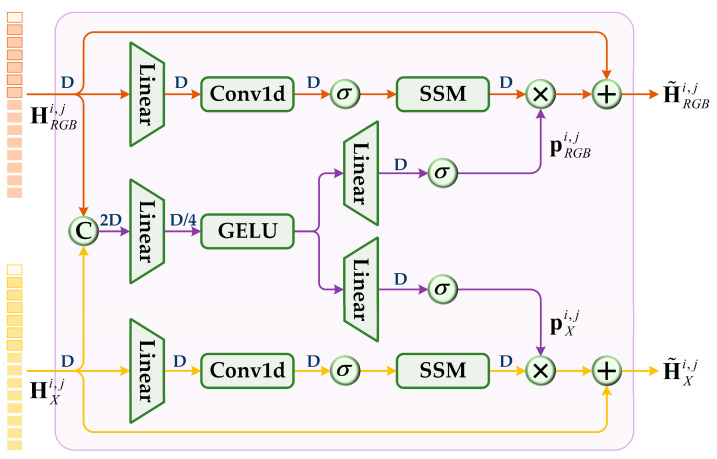
Details of the mamba synergy prompt block (MSPB). SSM, state space model. σ denotes SiLU activation function.

As shown in Figure 4, MSPB adopts two parallel mamba branches to enhance the RGB and X modality features at each encoding stage. The output features $H_{bm}^{i,j}=[H_{RGB}^{i,j};H_{X}^{i,j}]$, j∈[1,2,3] from each encoding stage are separated into RGB and X modalities $H_{RGB}^{i,j}$, $H_{X}^{i,j}$ via batch splitting and fed into MSPB. In MSPB, the two modal features are first concatenated along the embedding dimension. This is followed by a reduction–expansion bottleneck (REB) structure that eliminates redundant and unreliable information and extracts modality-specific prompts $p_{RGB}^{i,j}$, $p_{X}^{i,j}\in {\mathbb{R}}^{N\times D}$,

(7) $$p_{RGB}^{i,j},p_{X}^{i,j}=\phi_{REB}^{j}\left(Concat\left(H_{RGB}^{i,j},H_{X}^{i,j}\right)\right)$$

where $\phi_{REB}^{j}\left(\cdot \right)$ represents REB structure operation. Next, $H_{RGB}^{i,j}=[\tau_{RGB}^{i,j},T_{RGB}^{i,j},S_{RGB}^{i,j}]$ and $H_{X}^{i,j}=[\tau_{X}^{i,j},T_{X}^{i,j},S_{X}^{i,j}]$ enter the main branches with the state space model (SSM) to capture long-range dependencies from all token sequences, and then multiply with the corresponding modality-specific prompts. Meanwhile, residual connections are introduced to retain the key information of the original features. The above process can be formalized as

(8) $${\tilde{H}}_{RGB}^{i,j}=H_{RGB}^{i,j}+p_{RGB}^{i,j}⊗M_{RGB}^{j}\left(H_{RGB}^{i,j}\right),$$

(9) $${\tilde{H}}_{X}^{i,j}=H_{X}^{i,j}+p_{X}^{i,j}⊗M_{X}^{j}\left(H_{X}^{i,j}\right)$$

where $M_{RGB}^{j}$ and $M_{X}^{j}$ represent the operations for the RGB and X modality branches, respectively, before introducing the prompt. Perform batch merging on enhanced modality features ${\tilde{H}}_{RGB}^{i,j}=[{\tilde{\tau }}_{RGB}^{i,j},{\tilde{T}}_{RGB}^{i,j},{\tilde{S}}_{RGB}^{i,j}]$ and ${\tilde{H}}_{X}^{i,j}=[{\tilde{\tau }}_{X}^{i,j},{\tilde{T}}_{X}^{i,j},{\tilde{S}}_{X}^{i,j}]$ as input for the next encoding stage. Obviously, MSPB ensures the intra- and inter-modality information exchange across spatial and temporal dimensions.

### 3.4. Gated Fusion and Context Awareness

**Figure 5:**
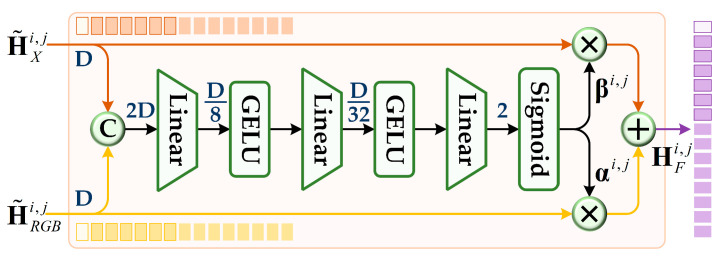
Schematic diagram of the gated fusion unit (GFU).

To achieve deep fusion between enhanced RGB and X modality features, we introduce a gated fusion unit (GFU) and a context awareness unit (CAU). GFU jointly processes features from both modalities, learning multi-modal weight factors to perform adaptive fusion on each token sequence. This design allows the model to dynamically modulate modality contributions and adaptively select critical information, significantly improving the discriminability and robustness of multi-modal representation. As depicted in Figure 5, GFU first concatenates the input features of the two modalities along the embedding dimension. Then, it progressively reduces dimensionality through multiple linear layers, obtains weight factors $\alpha^{i,j}$, $\beta^{i,j}\in {\mathbb{R}}^{N\times 1}$ via the sigmoid activation function, and finally performs weighted fusion to generate multi-modal fusion feature $H_{F}^{i,j}$.

(10) $$H_{F}^{i,j}=\alpha^{i,j}⊗{\tilde{H}}_{RGB}^{i,j}+\beta^{i,j}⊗{\tilde{H}}_{X}^{i,j}$$

**Figure 6:**
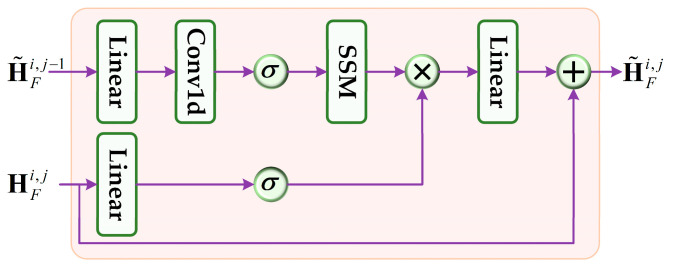
Structure of the context awareness unit (CAU). σ denotes SiLU activation function.

Furthermore, CAU progressively incorporates low-level fusion features rich in detailed information to improve the expressive capacity of high-level fusion features. As shown in Figure 6, in CAU, the low-level fusion feature $H_{F}^{i,j-1}$ is fed into a branch containing an SSM to model long-range dependencies for all fusion token sequences. Concurrently, the high-level feature $H_{F}^{i,j}$ enters another branch containing a linear layer and an SiLU activation layer. Next, element-wise multiplication is performed on the outputs of these two branches, utilizing the high-level feature as guidance to enhance key information and suppress noise in the low-level feature. Additionally, a residual connection is introduced in CAU. The final fusion feature ${\tilde{H}}_{F}^{i}=[\tau_{F}^{i},{\tilde{T}}_{F}^{i},{\tilde{S}}_{F}^{i}]$ of the *i*-th frame is obtained after the last CAU, which is then input into the TGAM and tracking head for prediction. In SMUTrack, this hierarchical fusion strategy enables the fusion feature with both local details and global context, improving tracking performance.

## 4. Experiments and Results

In this section, we first detail the training and inference pipeline of the proposed SMUTrack, then compare it with state-of-the-art trackers on several mainstream MMOT datasets. Finally, we conduct an ablation study of its core modules, substantiating the effectiveness and superiority of SMUTrack.

### 4.1. Implementation Settings

We adopt the same tracking prediction head and loss function as OSTrack [2]. The transformer visual encoder employs ViT-Base [37] architecture, and its parameters are initialized by MAE [38] pre-training parameters. SMUTrack is built on the PyTorch 1.13.1 platform and trained on three types of multi-modal image pairs, including RGB-D, RGB-T, and RGB-E, with data sourced from DepthTrack [39], LasHeR [40], and VisEvent [23] datasets, respectively. To avoid biasing the training model towards a specific modality type, the number of RGB-T, RGB-D, and RGB-E samples in each training batch is always consistent. For the input data, we use three 128 × 128 template image pairs and two 256 × 256 search image pairs. The sampling interval of the video sequence is set to 400, which better approximates the entire video content and captures the long-term motion changes in the tracked object. The training process is carried out on six NVIDIA GeForce RTX 3090 GPUs over a total of 60 epochs. Each epoch contains 60,000 image pairs, and the batch size is set to nine. The optimizer uses AdamW [41] with a weight decay of 10^−4^. For the initial learning rate, the backbone network is set to 4 × 10^−5^, and other parameters are set to 4 × 10^−4^. The learning rate decays by a factor of 10 after 48 epochs.

The inference stage is consistent with the training setting, and three template frames are selected using a dynamic template update strategy. The proposed SMUTrack has 102.83 M parameters and 67.87 G FLOPs. On a GeForce RTX 4090 GPU, the average speed in LasHeR is approximately 41 FPS.

### 4.2. Comparison with State-of-the-Arts

We evaluate the proposed SMUTrack against state-of-the-art trackers on five benchmark datasets across three downstream MMOT tasks.

#### 4.2.1. Comparison on RGB-D Datasets

DepthTrack [39] is a large-scale long-term RGB-D tracking benchmark, which contains 150 training and 50 testing video sequences, featuring 15 per-frame attributes and an average sequence length of 1473. It uses precision (Pr), recall (Re), and F-score as evaluation metrics. Table 3 compares SMUTrack with 25 previous state-of-the-art trackers. SMUTrack exhibits the best performance, achieving 63.9% (F-score), 63.8% (recall), and 64.0% (precision).

VOT-RGBD2022 [53] comprises 127 short-term RGB-D sequences. It adopts an anchor-based evaluation protocol [49] that requires trackers to undergo multiple initialization starts from different points. As reported in Table 3, the evaluation metrics encompass accuracy (Acc.), robustness (Rob.), and expected average overlap (EAO). SMUTrack obtains the highest scores on these metrics, with values of 93%, 82.0%, and 76.9%, respectively; it has a 2.5% improvement over SeqTrackv2 in EAO.

#### 4.2.2. Comparison on RGB-T Datasets

**Figure 7:**
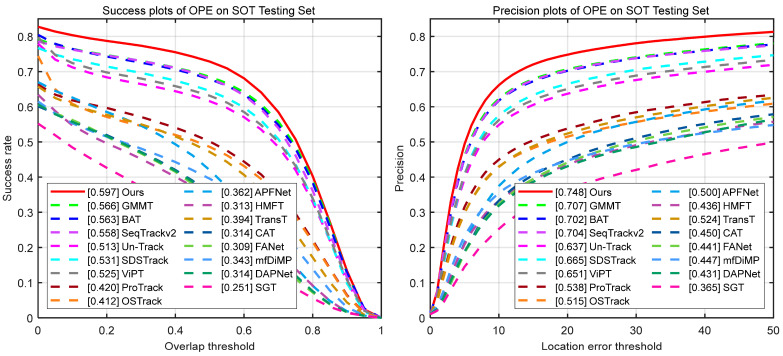
Success rate curves (**left**) and precision rate curves (**right**) on the LasHeR testing set.

**Figure 8:**
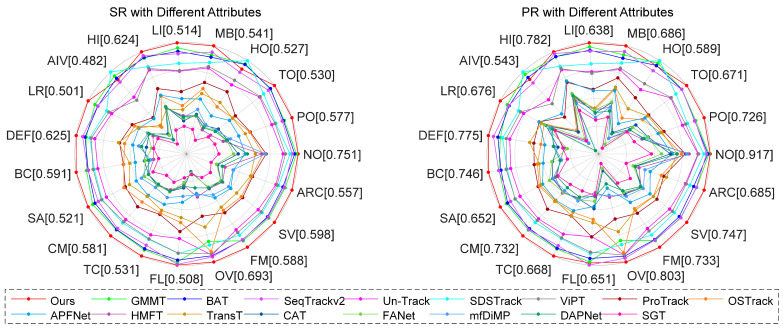
Comprehensive comparison under 19 different attributes on the LasHeR testing set. NO, No Occlusion; PO, Partial Occlusion; TO, Total Occlusion; HO, Hyaline Occlusion; MB, Motion Blur; LI, Low Illumination, HI, High Illumination; AIV, Abrupt Illumination Variation; LR, Low Resolution; DEF, Deformation; BC, Blackground Clutter; SA, Similar Appearance; CM, Camera Moving; TC, Thermal Crossover; FL, Frame Lost; OV, Out of View; FM, Fast Motion; SV, Scale Variation; ARC, Aspect Ratio Change.

LasHeR [40] comprises 1224 aligned RGB-T video sequences (978 for training, 244 for testing), totaling 730 K image pairs. This dataset covers 32 categories and 19 challenging attributes. Evaluation metrics include precision rate (PR), success rate (SR), and normalized precision rate (NPR). As illustrated in Table 4 and Figure 7, SMUTrack achieves the top performance in quantitative evaluation metrics and curves. We further analyze the capabilities of trackers in various challenging attributes, including illumination variation, occlusion, motion blur, etc. As shown in Figure 8, our SMUTrack performs extremely well in these extreme scenarios and demonstrates remarkable robustness. Quantitatively, SMUTrack outperforms the suboptimal GMMT by 3.4% under partial occlusion and 2.1% under total occlusion.

**Figure 9:**
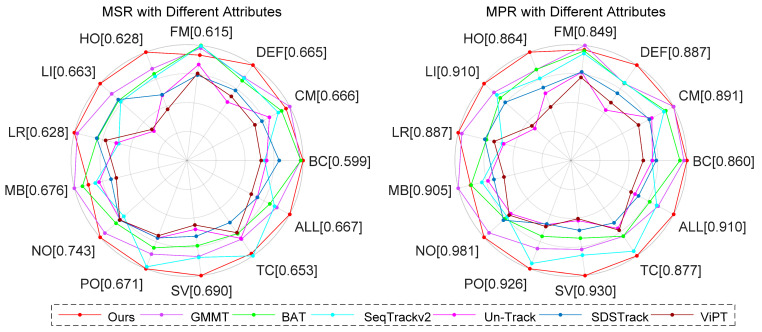
Performance comparison under 12 different attributes on the RGBT234 dataset. NO, No Occlusion; PO, Partial Occlusion; HO, Heavy Occlusion; LI, Low Illumination; LR, Low Resolution; TC, Thermal Crossover; DEF, Deformation; FM, Fast Motion; SV, Scale Variation; MB, Motion Blur; CM, Camera Moving; BC, Background Clutter; ALL, All Attributes.

RGBT234 [65] encompasses 234 video sequences carrying 12 annotated attributes. Maximum precision rate (MPR) and maximum success rate (MSR) are adopted as evaluation metrics, which consider the alignment error problem. In Table 4, our SMUTrack obtains the optimal performance with 66.7% MSR and 91% MPR, outperforming GMMT by 1.7% and 2.7%, respectively. Moreover, it adapts well to most challenging scenarios, as illustrated in Figure 9.

#### 4.2.3. Comparison on RGB-E Datasets

**Figure 10:**
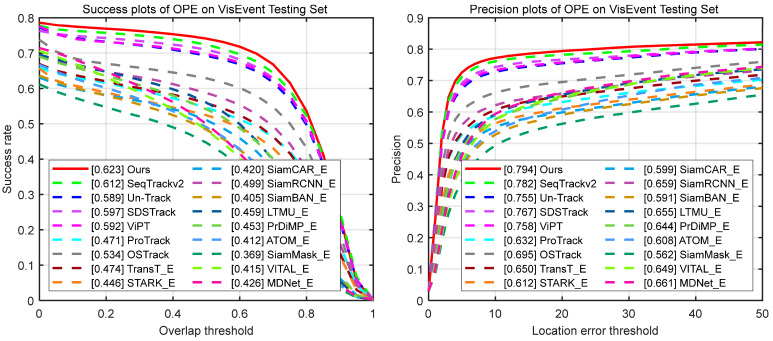
Overall performance on the VisEvent testing set.

**Figure 11:**
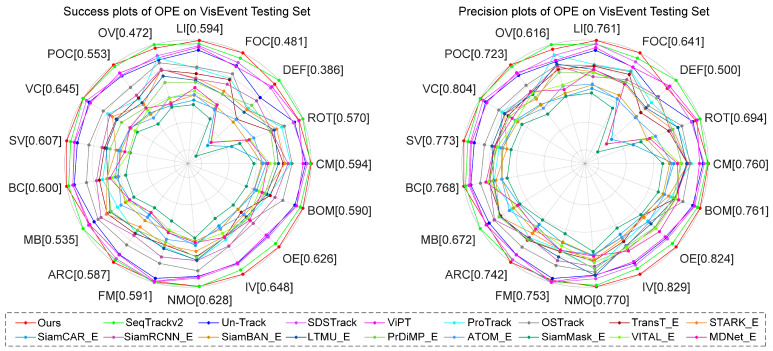
Performance comparison under 17 different attributes on the VisEvent dataset. CM, Camera Motion; ROT, Rotation; DEF, Deformation; FOC, Full Occlusion; LI, Low Illumination; OV, Out of View; POC, Partial Occlusion; VC, Viewpoint Change; SV, Scale Variation; BC, Background Clutter; MB, Motion Blur; ARC, Aspect Ration Change; FM, Fast Motion; NMO, No Motion; IV, Illumination Variation; OE, Over Exposure; BOM, Background Object Motion.

VisEvent [23] comprises 820 sequences (500 used for training, 320 for testing). It utilizes conventional SR, PR, and NPR metrics to evaluate tracker performance. We compare SMUTrack with 18 state-of-the-art trackers. As illustrated in Table 5 and Figure 10, our SMUTrack has the best overall performance in quantitative evaluation metrics and curves. Moreover, it demonstrates strong capability in handling tracking tasks across diverse challenging scenarios (Figure 11). Notably, in partial occlusion and full occlusion scenarios, SMUTrack outperforms the suboptimal SeqTrackv2 by 0.8% and 2.1%, respectively.

### 4.3. Ablation Study

To validate the effectiveness of each module in SMUTrack, we perform an ablation study on DepthTrack, LasHeR, and VisEvent datasets, with detailed results presented in Table 6. The baseline model utilizes ViT as the visual encoder to extract RGB and X modality features. These features are fused through element-wise addition before being fed into the prediction head. To tackle the inadequate utilization of object spatial–temporal information, we designed the SIP module. This module effectively captures object appearance changes and facilitates temporal information flow across frames through multi-template updating and temporal prompt learning. Compared to the baseline, integrating the SIP module improves the average performance of the tracker by 3.3%. Considering the importance of RGB and X modality information interactions for the perception ability of the model, we further introduce the HMSR module. By embedding MSPB into the three encoding stages of ViT, we enhanced the representation learning capacity of the model, leading to an additional 1.3% improvement in tracking performance. Furthermore, to achieve deep fusion of RGB and X modality features, we designed a GFCA module; it regulates inter-modal information weights via a gated fusion unit (GFU), captures the correlations of cross-level fusion features in conjunction with a context awareness unit (CAU), and learns a multi-modal representation with global context. The GFCA module contributes to a further 0.2% enhancement in tracking accuracy.

We further validate the effectiveness of the two types of subcomponents in the GFCA module, with the results presented in Table 7. Specifically, when the multi-level fusion features obtained from GFUs are simply added together, tracking performance on the DepthTrack dataset deteriorates significantly (Experiment 2 in Table 7). The main reason is that low-level fusion features contain richer details but also come with certain noise. Especially, depth modality exhibits more inconsistent quality and blurred details compared to other modalities, making the model more sensitive to such noise. When multi-modal features from different levels are fused through direct addition and the contextual information is aggregated solely via CAUs, the overall model performance shows an obvious decline (Experiment 3 in Table 7). This result indicates that the additive approach introduces more redundant information and noise, negatively impacting the contextual aggregation process. This also reveals the importance of adaptive fusion within GFUs. GFUs and CAUs work synergistically to form an effective complementarity, enabling the model to achieve relatively superior performance (Experiment 4 in Table 7). Additionally, each GFU has 0.15 M parameters and 0.067 G FLOPs, while each CAU has 1.872 M parameters and 0.823 G FLOPs, with negligible impact on model complexity and computational efficiency.

### 4.4. Visualization Results

**Figure 12:**
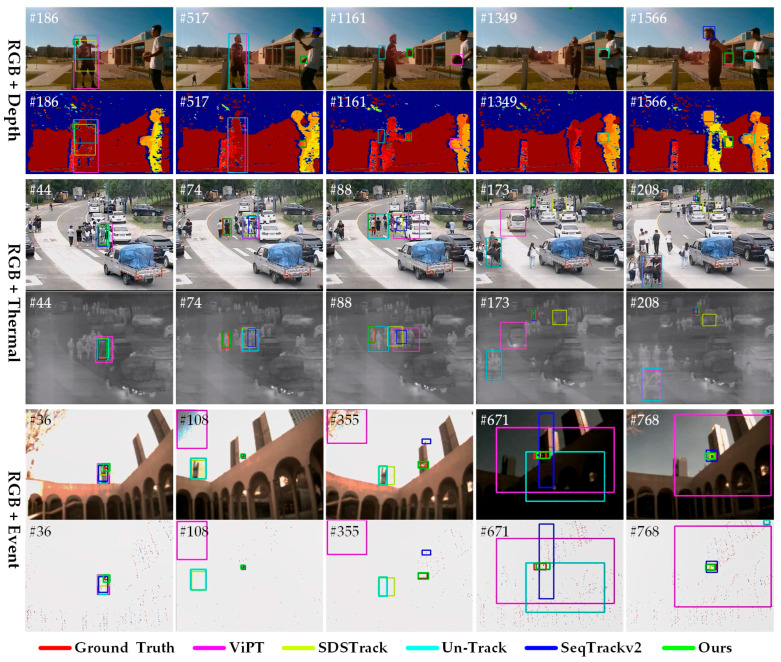
The visual comparison of our SMUTrack with the other four unified multi-modal trackers in three multi-modal tasks.

In Figure 12, we present a qualitative comparison between SMUTrack and four of the latest unified multi-modal trackers including ViPT [25], SDSTrack [26], Un-Track [29], and SeqTrackv2 [30]. These instances cover three combined modalities and diverse challenge attributes. In RGB-D video sequences, SMUTrack achieves stable and precise long-term object tracking even when encountering challenges such as partial occlusion and similar appearances. For RGB-T sequences, the tracked object is a composite of a human and a bicycle, with the main body severely occluded by dense pedestrians and vehicles as it rapidly moves from near to far. In this case, SMUTrack still achieves robust tracking by leveraging historical trajectory information. In RGB-E sequences, SMUTrack integrates RGB appearance features with event flow motion detail features. When confronting challenges such as low-resolution imaging, illumination variations, and complex backgrounds, it demonstrates excellent performance, enabling precise continuous tracking of an unmanned aerial vehicle. Overall, SMUTrack effectively exploits the complementary relationship between the RGB modality and arbitrary X modality and fully leverages the spatial–temporal information of video sequences, thereby significantly enhancing the perceptual and discriminative capacities of the tracking model for the object and its environmental context.

## 5. Conclusions

In this work, we propose SMUTrack, a unified MMOT framework featuring a unique network architecture and a unique parameter set. By leveraging the latent relationships among intra- and inter-task modalities, as well as the spatial–temporal intrinsic correlations in video sequences, SMUTrack effectively mitigates cross-modal inherent heterogeneity and inter-modal synergy imbalance, and its spatial–temporal perception and representation learning abilities are significantly improved. SMUTrack achieves excellent performance in three downstream MMOT tasks, exhibiting strong generalization capability. Extensive experiments validate the superiority and robustness of the proposed framework. Future work will focus on integrating the powerful representation capabilities of large models into the multi-modal tracking model while pursuing the lightweight design to promote precise understanding and efficient deployment in more challenging open-world scenarios.

## Figures and Tables

**Table 1:** Overview of related work on multi-modal object tracking.

Category	Related Works	Data Types	Descriptions
Task-specific	AMATrack [27]	RGB-D	Designed for a specific RGB-X task, they may excel at one type of task but fail to consider potential modality relationships across tasks.
	AMNet [17]	RGB-T	
	GMMT [28]	RGB-T	
	RT-MDNet [23]	RGB-E	
Task-general	ViPT [25]	RGB-D, RGB-T, RGB-E	Only a few prompts are needed for learning, but modality synergy and generalization capabilities still need improvement.
	SDSTrack [26]		
	Un-Track [29]		

**Table 2:** Overview of related work on spatial–temporal modeling.

Category	Related Works	Descriptions
Updating appearance representations	SeqTrack [30]	Online template update strategies are typically employed to capture real-time appearance changes, yet sparse template updates struggle to establish stable spatial–temporal correlations.
	STARK [31]	
	PromptVT [32]	
	TABBTrack [33]	
Modeling temporal dependencies	ARTrack [3]	Learning a global temporal representation across video sequences enables effective perception of trajectory evolution processes.
	AQATrack [34]	
	TCTrack [36]	

**Table 3:** State-of-the-art comparisons on RGB-D datasets. **Red** and **green** indicate the best and second best performances.

Method	Source	VOT-RGBD22			DepthTrack		
		EAO	Acc.	Rob.	F-Score	Re	Pr
SMUTrack	Ours	**76.9**	**82.0**	**93.0**	**63.9**	**63.8**	**64.0**
SeqTrackv2 [30]	CVPR’24	**74.4**	81.5	**91.0**	**63.2**	**63.4**	**62.9**
OneTracker [42]	CVPR’24	72.7	**81.9**	87.2	60.9	60.4	60.7
Un-Track [29]	CVPR’24	71.8	**82.0**	86.4	61.0	61.0	61.0
SDSTrack [26]	CVPR’24	72.8	81.2	88.3	61.4	60.9	61.9
SPT [14]	AAAI’23	65.1	79.8	85.1	53.8	54.9	52.7
ViPT [25]	CVPR’23	72.1	81.5	87.1	59.4	59.6	59.2
ProTrack [43]	ACMMM’22	65.1	80.1	80.2	57.8	57.3	58.3
OSTrack [2]	ECCV’22	67.6	80.3	83.3	52.9	52.2	53.6
SBT-RGBD [44]	CVPR’22	70.8	80.9	86.4	-	-	-
DMTracker [13]	ECCV’22	65.8	75.8	85.1	-	-	-
DeT [39]	ICCV’21	65.7	76.0	84.5	53.2	50.6	56.0
STARK-RGBD [31]	ICCV’21	64.7	80.3	79.8	-	-	-
KeepTrack [45]	ICCV’21	60.6	75.3	79.7	-	-	-
DRefine [46]	ICCV’21	59.2	77.5	76.0	-	-	-
DAL [47]	ICPR’21	-	-	-	42.9	36.9	51.2
TSDM [48]	ICPR’21	-	-	-	38.4	37.6	39.3
DDiMP [49]	ECCV’20	-	-	-	48.5	46.9	50.3
ATCAIS [49]	ECCV’20	55.9	76.1	73.9	47.6	45.5	50.0
LTMU-B [50]	CVPR’20	-	-	-	46.0	41.7	51.2
GLGS-D [49]	ECCV’20	-	-	-	45.3	36.9	58.4
Siam-LTD [49]	ECCV’20	-	-	-	37.6	34.2	41.8
LTDSEd [51]	ICCVW’19	-	-	-	40.5	38.2	43.0
SiamM-Ds [51]	ICCVW’19	-	-	-	33.6	26.4	46.3
DiMP [1]	ICCV’19	54.3	70.3	73.1	-	-	-
ATOM [52]	CVPR’19	50.5	69.8	68.8	-	-	-

**Table 4:** State-of-the-art comparisons on RGB-T datasets. **Red** and **green** indicate the best and second best performances.

Method	Source	LasHeR			RGBT234	
		SR	PR	NPR	MSR	MPR
SMUTrack	Ours	**59.7**	**74.8**	**71.3**	**66.7**	**91.0**
GMMT [28]	AAAI’24	**56.6**	**70.7**	67.0	**65.0**	**88.3**
BAT [24]	AAAI’24	56.3	70.2	66.4	64.1	86.8
TATrack [54]	AAAI’24	56.1	70.2	66.7	64.4	87.2
SeqTrackv2 [30]	CVPR’24	55.8	70.4	**67.2**	64.7	88.0
OneTracker [42]	CVPR’24	53.8	67.2	-	64.2	85.7
Un-Track [29]	CVPR’24	51.3	63.7	60.1	62.5	84.2
SDSTrack [26]	CVPR’24	53.1	66.5	62.7	62.5	84.8
TBSI [55]	CVPR’23	55.6	69.2	65.7	63.7	87.1
ViPT [25]	CVPR’23	52.5	65.1	61.7	61.7	83.5
ProTrack [43]	ACMMM’22	42.0	53.8	49.8	59.9	79.5
OSTrack [2]	ECCV’22	41.2	51.5	48.2	54.9	72.9
APFNet [56]	AAAI’22	36.2	50.0	43.9	57.9	82.7
HMFT [57]	CVPR’22	31.3	43.6	38.1	-	-
TransT [58]	CVPR’21	39.4	52.4	48.0	-	-
CMPP [59]	CVPR’20	-	-	-	57.5	82.3
CAT [60]	ECCV’20	31.4	45.0	39.5	56.1	80.4
FANet [61]	TIV’20	30.9	44.1	38.4	55.3	78.7
mfDiMP [62]	ICCVW’19	34.3	44.7	39.5	42.8	64.6
DAPNet [63]	ACMMM’19	31.4	43.1	38.3	-	-
SGT [64]	ACMMM’17	25.1	36.5	30.6	47.2	72.0

**Table 5:** State-of-the-art comparisons on RGB-E datasets. ‘_E’ represents the extension of RGB trackers with event fusion. **Red** and **green** indicate the best and second best performances.

Method	Source	VisEvent		
		SR	PR	NPR
SMUTrack	Ours	**62.3**	**79.4**	**75.4**
SeqTrackv2 [30]	CVPR’24	**61.2**	**78.2**	**73.9**
OneTracker [42]	CVPR’24	60.8	76.7	-
Un-Track [29]	CVPR’24	58.9	75.5	71.0
SDSTrack [26]	CVPR’24	59.7	76.7	72.3
ViPT [25]	CVPR’23	59.2	75.8	71.5
ProTrack [43]	ACMMM’22	47.1	63.2	56.0
OSTrack [2]	ECCV’22	53.4	69.5	64.6
TransT_E [58]	CVPR’21	47.4	65.0	58.3
STARK_E [31]	ICCV’21	44.6	61.2	53.7
SiamCAR_E [66]	CVPR’20	42.0	59.9	52.8
SiamRCNN_E [67]	CVPR’20	49.9	65.9	60.6
LTMU_E [50]	CVPR’20	45.9	65.5	57.0
PrDiMP_E [68]	CVPR’20	45.3	64.4	55.5
SiamBAN_E [69]	CVPR’20	40.5	59.1	50.7
ATOM_E [52]	CVPR’19	41.2	60.8	50.8
SiamMask_E [70]	CVPR’19	36.9	56.2	47.1
VITAL_E [71]	CVPR’18	41.5	64.9	52.9
MDNet_E [72]	CVPR’16	42.6	66.1	55.7

**Table 6:** Quantitative results of ablation studies on DepthTrack, LasHeR, and VisEvent datasets. Δ denotes the average value of performance change compared to the benchmark. **Red** is the best performance.

Components				DepthTrack			LasHeR			VisEvent			Δ
Baseline	SIP	HMSR	GFCA	F-Score	Re	Pr	SR	PR	NPR	SR	PR	NPR	
√				58.3	58.7	58.0	53.8	67.0	63.5	61.1	77.9	74.1	-
√	√			63.2	63.4	63.0	57.5	72.4	68.8	61.2	78.1	74.3	3.3
√	√	√		**64.3**	**64.3**	**64.4**	59.5	74.6	71.0	61.9	78.3	74.9	1.3
√	√	√	√	63.9	63.8	64.0	**59.7**	**74.8**	**71.3**	**62.3**	**79.4**	**75.4**	0.2

√ denotes the presence of the corresponding component.

**Table 7:** Ablation studies in the GFCA module. Δ denotes the average value of performance change compared to benchmark 1.

Number	GFU	CAU	DepthTrack			LasHeR			VisEvent			Δ
			F-Score	Re	Pr	SR	PR	NPR	SR	PR	NPR	
1	×	×	64.3	64.3	64.4	59.5	74.6	71.0	61.9	78.3	74.9	-
2	√	×	61.9	62.0	61.9	59.5	74.8	71.2	62.2	79.4	75.3	−0.6
3	×	√	62.6	62.2	62.9	59.2	74.5	70.6	61.2	78.0	74.2	−0.9
4	√	√	63.9	63.8	64.0	59.7	74.8	71.3	62.3	79.4	75.4	0.2

√ and × denote the presence or absence of the corresponding component.

## Data Availability

The original contributions presented in this study are included in the article. Further inquiries can be directed to the corresponding author.
